# Nestling birds learn socially to eavesdrop on heterospecific alarm calls through acoustic association

**DOI:** 10.1098/rspb.2025.0879

**Published:** 2025-07-16

**Authors:** Jinggang Zhang, Chenyang Zhao, Peter Santema, Zixuan Lin, Jianqiang Li, Wenhong Deng, Bart Kempenaers

**Affiliations:** ^1^ Ministry of Education Key Laboratory for Biodiversity Sciences and Ecological Engineering, College of Life Sciences, Beijing Normal University, Beijing 100875, People's Republic of China; ^2^ Department of Biology, Faculty of Arts and Sciences, Beijing Normal University at Zhuhai, Zhuhai, 519087, People's Republic of China; ^3^ Department of Ornithology, Max Planck Institute for Biological Intelligence, 82319 Seewiesen, Germany; ^4^ Edward Grey Institute, Department of Biology, University of Oxford, Oxford OX1 3PS, UK; ^5^ School of Ecology and Nature Conservation, Beijing Forestry University, Beijing, 100083, People's Republic of China

**Keywords:** nestlings, Daurian redstart, alarm call, nest predation, social learning, acoustic association

## Abstract

Animals often eavesdrop on signals intended for others to gather information about their environment. While adult animals have been shown to learn to recognize unfamiliar heterospecific alarm calls through both social and asocial learning, it remains unclear whether and how young animals learn to recognize unfamiliar alarm calls. We show experimentally that nestling Daurian redstarts, *Phoenicurus auroreus*, can socially learn to recognize unfamiliar heterospecific alarm signals by associating them with conspecific alarm calls. We trained nestlings by presenting two unfamiliar sounds, one together with conspecific alarm calls (training) and one without (control). Before training, nestlings showed similarly little response to both novel sounds. After training, however, nestlings showed clear anti-predator responses to the training sound, but not to the control sound. These results show that nestling birds can socially learn to associate novel sounds with known alarm calls, even without visual confirmation of danger.

## 1. Introduction

Predation is a major source of mortality for many species, and animals often use alarm calls to warn each other of danger [1,2]. Variations in the acoustic structure of alarm calls often encode detailed information about the predator, such as its type, behaviour, size, distance and threat level [1,3–6]. These calls are not only used by conspecifics but are also eavesdropped on by other species [7–9]. Eavesdropping on heterospecific alarm calls allows receivers to gain valuable information about predators, improving their survival chances and ability to respond appropriately to different types of predatory threats [10–13]. This behaviour also enables naive individuals to learn about new predators and the risks they pose [14–17].

Responses to heterospecific alarm calls can be innate or learned. Innate recognition of heterospecific alarm calls may arise when such calls share acoustic features with familiar calls or have general alerting properties like harshness [7,18–23]. However, there is substantial variation in alarm calls among species, which may limit the effectiveness of innate responses, emphasizing the role of learning in facilitating eavesdropping across taxa [7]. Individuals can learn to eavesdrop on heterospecific alarm calls through personal experience, whereby the individuals form direct associations between predators and alarm signals [24]. Although direct learning avoids errors from copying the behaviour of others, it can be risky if it involves exposure to predators [25]. Alternatively, social learning allows individuals to associate heterospecific alarm calls with the fearful responses of demonstrators, without direct predator exposure [25–28]. Experimental evidence has demonstrated that individuals can learn to eavesdrop on unfamiliar alarm calls both directly, by associating the novel sounds with predator models [17], and socially, by associating them with known alarm calls [16]. However, most studies investigating learning mechanisms focus on adults [15–17,29–34]. Whether and how young animals learn to eavesdrop on heterospecific alarm calls remains largely unclear [19,35].

When exposed to predators, adult birds and young that have already fledged can actively flee, while nestlings that are not yet ready to fledge are simply passive victims of predation. Therefore, it is particularly important for nestlings to be aware of predators and respond in a way that avoids being detected (e.g. going silent). Given the general high risk of nest predation [36], nestlings should be under strong selection to develop immediate responses to cues indicating danger, such as heterospecific alarm calls [37,38]. However, empirical evidence on whether nestlings can learn to recognize heterospecific alarm calls remains controversial [19,35], and the underlying mechanisms of learning remain unknown.

Here, we experimentally test whether nestlings of free-living Daurian redstarts *Phoenicurus auroreus*, a passerine bird widely distributed in China, can socially learn to recognize unfamiliar heterospecific alarm calls. Redstarts are cavity-nesting breeders. Thus, unlike nestlings of open-nesting species, which can assess predation risk through visual cues (e.g. by spotting the predator), redstart nestlings have a limited field of view to observe the environment outside their nests. It is therefore crucial for them to rely on auditory cues, such as alarm calls from conspecifics or heterospecifics, to assess predation risk. In the field, when a raptor such as the Eurasian sparrowhawk, *Accipiter nisus*, flies overhead, adult Daurian redstarts typically flee to cover and remain silent, while sympatric species such as white wagtails (*Motacilla alba*), barn swallows (*Hirundo rustica*) and red-rumped swallows (*Cecropis daurica*), mob the predator in a group with aerial alarm calls (personal observations by J.Z., C.Z., and Z.L. during each field season between 2018 and 2024). Because the parents are not always near the nest to warn of danger [37,39,40], eavesdropping on heterospecific alarm calls can help nestlings assess predation risk and respond proactively to reduce the likelihood of being detected (e.g. stopping begging). The ability to learn to recognize heterospecific alarm calls may thus be critical for nestling survival.

We hypothesized that Daurian redstart nestlings can learn to recognize unfamiliar alarm calls by associating them with known alarm calls, in the absence of any visual cues from callers or predators. To test this hypothesis, we first exposed the nestlings to two unfamiliar sounds, i.e. a brown-flanked bush warbler (*Horornis fortipes*; hereafter, warbler) song and a common rosefinch (*Carpodacus erythrinus*; hereafter, rosefinch) song, to test their innate response to these sounds (pre-training test). Next, we trained the nestlings with the two sounds: in the warbler group, the warbler sound was broadcasted six times together with conspecific alarm calls (training sound), and the rosefinch sound was broadcasted six times without conspecific alarm calls (control sound); in the rosefinch group, the rosefinch sound was broadcasted six times together with conspecific alarm calls (training sound), and the warbler sound was broadcasted six times without conspecific alarm calls (control sound). After the training, we re-exposed the nestlings to the two sounds to assess changes in their responses to the novel sounds (i.e. lowering of the head and cessation of begging) before and after training. Based on the hypothesis, we predicted that after training nestlings would be more likely to respond to the training sound, but not to the control sound.

## 2. Methods

### (a) Study system and general procedures

The study was conducted in ShuangYu, a village in northeastern China (43°37′19″N, 126°09′54″E) in 2024. Daurian redstarts typically build their nests in concealed sites such as cavities in trees, walls and eaves, and on the ground, and they readily use artificial nest boxes [41]. Between 2016 and 2022, we placed 240 nest boxes in the study site, with a distance between adjacent boxes of approximately 50 m. In the study area, the redstarts produce on average five chicks per brood (brood size (mean ± s.d.): 5.3 ± 1.3 (*n* = 95)).

During the breeding season, we searched for natural nests daily and checked nest boxes weekly. In the late incubation stage, we visited the nests every day to determine the hatching date. We conducted the playback experiments on the brood when the nestlings were 11−13 days old, which is shortly before the expected fledging day (typically at 14 days old).

### (b) Playback stimuli

**Figure 1.**
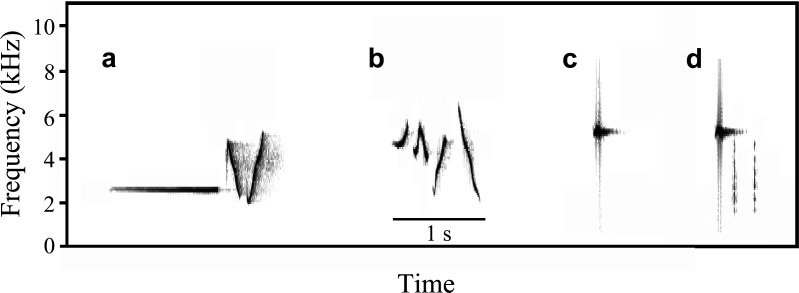
Sounds used in the playback experiment. Spectrograms of a single note from all sounds used in the experiment: brown-flanked bush warbler (a), common rosefinch (b) and Daurian redstart (c, d). Sp*e*ctrograms were produced in Avisoft-SASLab Lite software (Avisoft Bioacoustics, Berlin, Germany) with a Hamming window function and sample size of 256 bits and grid overlap 50% from recordings digitized at 22.05 kHz and 16 bits.

The aim of our experiment was to investigate whether nestlings were able to learn to recognize an unfamiliar sound by associating it with conspecific alarm calls. Given that alarm calls often share similar acoustic structures across species, individuals sometimes show innate responses to unfamiliar heterospecific alarm calls without learning [7,23]. To minimize the possibility of innate responses from redstart nestlings, we used songs from two species: the brown-flanked bush warbler and the common rosefinch. These species are widespread and common in China, so adult Daurian redstarts should be familiar with their sounds, such that they will not respond during the experiment. However, neither species is present in the study area during the breeding season, and their sounds differ from any known sounds of local species, which means that redstart nestlings will never have encountered the sounds. Additionally, both sounds are short and have a distinct structure (figure 1), making them easily distinguishable and suitable for editing. The novel sounds were downloaded from https://www.xeno-canto.org/, while Daurian redstart alarm calls were recorded in previous field seasons using a recorder (DR-05X, TASCAM). Daurian redstarts typically utter these alarm calls in response to moving predators, such as cats or snakes, near the nest. We edited playback stimuli using the software Audacity (https://www.audacityteam.org/). Each stimulus lasted about 30 s and contained 6−8 repeated elements. To avoid pseudoreplication, we used different recordings for each stimulus: 9 recordings for warblers, 9 for rosefinches, and 6 for Daurian redstart alarm calls. We calibrated the amplitude of each playback stimulus to a natural volume of 70 dB, measured 1 m from the speaker using a sound level meter (AS824, SMART SENSOR).

### (c) Playback trials

The experimental procedure involved repeated playbacks to redstart nestlings, which included a pre-training test to assess the initial response to these unfamiliar sounds, followed by repeated training sessions and then a post-training assessment to evaluate learning.

#### (i) Pre-training

The pre-training trials were aimed to assess the nestlings’ initial response to the two unfamiliar sounds, one of which was later used as the training sound and the other as the control. On the morning when the nestlings were 11 days old (around 7.30), we set up a video camera (SMX-F50, Samsung) about 1 m in front of the nest to allow the nestlings to habituate to the camera. After 1 h, we played back one unfamiliar sound (warbler or rosefinch), and after another 30 min the other sound (rosefinch or warbler). For playback, we used a portable speaker (BV200, See Me Here) placed about 3−5 m from the nest. We randomized the order of the playback of the two sounds. After the final playback, the camera continued recording for an additional 5 min to capture the nestlings’ response to the unfamiliar sounds, i.e. lowering of the head and cessation of begging.

#### (ii) Training

After the pre-training test, the nestlings were trained to associate one of the unfamiliar sounds with predator alarm by playing it back together with conspecific alarm calls (figure 1). Each brood was randomly assigned to either the warbler or rosefinch training group. In the warbler group, nestlings were trained to associate warbler sounds with conspecific alarm calls, while rosefinch sounds served as the control. In the rosefinch group, nestlings were trained to associate rosefinch sounds with conspecific alarm calls, with warbler sounds as the control.

One hour after the final pre-training playback (around 10.00), we exposed the nestlings to six training trials over the course of a single day. The interval between consecutive trials was 1 h, and each trial consisted of two playbacks: a training playback and a control playback. During the training playback, we played back the training sound together with conspecific alarm calls using two speakers. To enhance realism and minimize habituation, the two speakers were separated by 2−5 m, each at a distance of 3−5 m from the focal nest. We randomized the order of the two sounds within the training playback, with the interval between their onset being less than 2 s. We also randomized the order of the training and control playbacks within each trial, with a 30 min interval between the two playbacks.

#### (iii) Post-training

On the day following training (test day 1, when nestlings were 12 days old) and again on the next day (test day 2, when nestlings were 13 days old), we tested the response of the nestlings to both the training and the control sounds following the same procedures as in the pre-training test.

Pilot experiments showed that adult redstarts did not respond towards the two novel sounds, but they occasionally emitted alarm calls towards the experimenters, particularly when the environment around the nest was open. Therefore, to minimize possible disturbance from the parents, we hid behind a cover near the nest at least 5 min before the playback to observe the parents and nest, and we started the playback only when the parents had left the nest for at least 2 min.

### (d) Nestling responses

We assessed the nestlings’ response within the first 30 s after the start of the playback. We chose this short response period because (i) passerine nestlings typically return to their normal behaviour within 1 min after playback [42], and (ii) redstart parents typically provision the nestlings frequently, and we excluded trials during which the parents returned to the nest (see below). Therefore, using a short response period maximized the effective sample size. Nestlings typically go silent when they sense danger [19,43], i.e. they suppress begging calls and movements, and crouch down inside the nest, to reduce the risk of being detected by predators (electronic supplementary material, videos S1 and S2) [35]. Therefore, we evaluated the nestlings’ response to playbacks based on whether they (i) emitted begging calls and (ii) lowered their heads. To account for potential observer bias, all video recordings were viewed independently by three people who were blind to the treatment that had been assigned to the broods.

We conducted the experiment on a total of 107 broods, but 12 were excluded from analyses: four broods were predated during the experiment and eight broods were excluded because the parents returned or emitted alarm calls during the experiment. Thus, the sample size for analyses was 95 broods (49 and 46 in the warbler and rosefinch training group, respectively).

### (e) Statistical analysis

To ensure that the scoring of the videos was not dependent on the interpretation of a particular observer, we assessed the repeatability of observations among the three observers. To this end, we used generalized linear mixed-effects models (GLMMs) with a binomial error structure, with nestling response as the dependent variable and observer identity and video-recording identity as random effects, using the R package *rptR* [44,45]. In the models, we included observer, group (warbler/rosefinch), stage (pre-training/test day 1/test day 2), nest type (natural/box nest), brood size and treatment (warbler/rosefinch) as fixed effects. Both observed responses showed a low repeatability for observer identity (the probability of begging: *r* = 0.002 [0, 0.01], *p* = 1; the probability of lowering head: *r* = 0.002 [0, 0.02], *p* < 0.001), indicating that the scores did not depend on the identity of the observer. In contrast, both responses showed a high repeatability for video-recording identity (the probability of begging: *r* = 0.886 [0.763, 0.976], *p* < 0.001; the probability of lowering head: *r* = 0.983 [0.926, 0.995], *p* < 0.001), indicating that scores of the same video by different observers were consistent. These analyses thus confirm that observations were indeed highly consistent across observers. Because only one value for each video recording should be included in the analyses, we randomly selected data from one observer for subsequent analyses.

We first tested whether the nestlings could learn to recognize unfamiliar sounds by comparing their responses to the sounds before and after training. To do this, we used generalized linear mixed models (GLMMs) with a binomial error structure, with nestling response (whether begging or lowering the head) as the dependent variable, nest identity as the random effect, and stage (pre-training/post-training (test day 1)), nest type (natural/box nest), brood size and treatment (warbler/rosefinch) as fixed effects. If the nestlings learned to recognize the unfamiliar sound, they should be more likely to respond to the training than to the control sound after training. Therefore, we also included the interaction between treatment and stage as a fixed effect. We then assessed the stability of the learned behaviour after training using GLMMs, with nestling response (whether begging or lowering the head) as the dependent variable, nest identity as the random effect, and stage (test day 1/test day 2), nest type (natural/box nest), brood size, treatment (warbler/rosefinch) and the interaction between treatment and stage as fixed effects. We analysed the warbler and rosefinch treatments separately, because these songs differ in acoustic properties (figure 1) and it is therefore plausible that nestlings learn better or respond more strongly to one novel sound compared with the other. To account for this possibility in one model would require a three-way interaction, but such interactions are difficult to interpret. Note that using a single model with pooled data did not affect the conclusions (electronic supplementary materials, tables S1 and S2).

All statistical analyses were carried out in R v. 4.1.1 [46]. The alpha level of the statistical tests was set at 0.05.

## 3. Results

**Figure 2.**
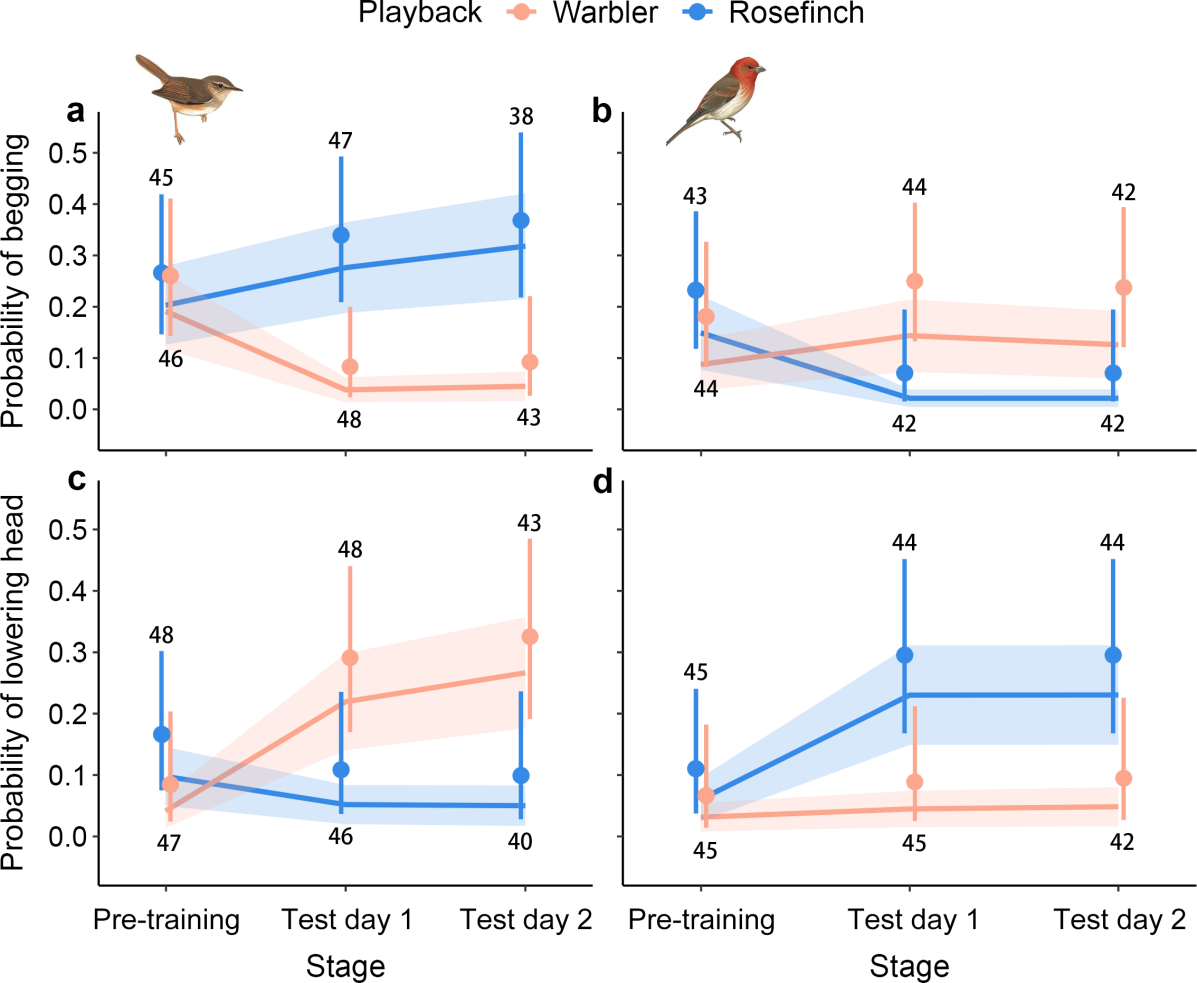
Variation in the probability of begging (a,b) and lowering the head (c,d) of Daurian redstart nestlings in the warbler (a,c) and rosefinch groups (b,d) before and after training*.* In the warbler group, nestlings were trained to associate warbler sounds with conspecific alarm calls, while rosefinch sounds were used as the control. In the rosefinch group, nestlings were trained to associate rosefinch sounds with conspecific alarm calls, with warbler sounds as the control. The lines and shaded areas represent model predictions and 95% confidence intervals. Data shown are means (dots) and 95% binomial confidence intervals (error bars) based on raw data. Numbers indicate sample size. Some broods were excluded from the pre-training data because parents returned to the nest during training, such that the sample size is lower during the pre-training than on test day 1 in (a) and (c). These broods were still included to assess the stability of learned behaviour.

Before training, only a few Daurian redstart nestlings responded to the unfamiliar sounds, and there was no difference in the probability of begging or lowering the head between nestlings exposed to warbler calls and those exposed to rosefinch calls (figure 2 and electronic supplementary material, table S3).

### (a) Probability of begging after training

For nestlings that were trained to the warbler sound (with associated alarm calls), there was a significant interaction effect between treatment (warbler/rosefinch) and stage (pre-training/post-training) (GLMM: estimate = 1.96, s.e. = 0.86, *z* = 2.28, *p* = 0.023; figure 2a and electronic supplementary material, model 1 in table S3). Nestlings were much less likely to make begging calls in response to the warbler sound after training compared with before training (GLMM: estimate = −1.58, s.e. = 0.68, *z* = −2.32, *p* = 0.020; figure 2a and electronic supplementary material, model 1 in table S3 and video S1). In contrast, they showed no difference in their begging propensity in response to the rosefinch sound (control treatment without associated alarm calls) after training compared with before training (GLMM: estimate = 0.37, s.e. = 0.51, *z* = 0.73, *p* = 0.47; figure 2a and electronic supplementary material, video S1). After training, nestlings were also less likely to beg in response to the warbler sound compared with the rosefinch sound (GLMM: estimate = −7.64, s.e. = 2.01, *z* = −3.80, *p* < 0.001; figure 2a and electronic supplementary material, model 5 in table S4). Nestlings did not show any variation in their begging propensity over the 2 days following training, as there was no interaction between the treatment and the day of the post-training test (GLMM: estimate = 0.26, s.e. = 1.73, *z* = 0.15, *p* = 0.88; figure 2a and electronic supplementary material, model 5 in table S4).

For nestlings that were exposed to the rosefinch sound (treatment with alarm calls), the interaction effect between treatment and stage was also significant (GLMM: estimate = 2.42, s.e. = 1.05, *z* = 2.31, *p* = 0.021; figure 2b and electronic supplementary material, model 2 in table S3). Nestlings were also less likely to produce begging calls in response to the rosefinch sound after training compared with before training (GLMM: estimate = −1.90, s.e. = 0.83, *z* = −2.31, *p* = 0.021; figure 2b and electronic supplementary material, model 2 in table S3). In contrast, they did not differ in their begging propensity when hearing the warbler sound (control treatment without associated alarm calls) after training compared with before training (GLMM: estimate = 0.51, s.e. = 0.62, *z* = 0.78, *p* = 0.44; figure 2b). After training, nestlings were less likely to beg in response to the rosefinch sound compared with the warbler sound (GLMM: estimate = −2.04, s.e. = 0.86, *z* = −2.37, *p* = 0.018; figure 2b and electronic supplementary material, model 6 in table S4). Moreover, there was no interaction between the treatment and the day of the post-training test (GLMM: estimate = −0.12, s.e. = 1.14, *z* = −0.11, *p* = 0.92; figure 2b and electronic supplementary material, model 6 in table S4), indicating that there was no variation in the nestlings’ begging propensity over the 2 days following training.

### (b) Probability of lowering the head after training

In the warbler group (treatment with alarm calls), there was a significant interaction effect between treatment and stage (GLMM: estimate = −2.82, s.e. = 1.10, *z* = −2.56, *p* = 0.011; figure 2c and electronic supplementary material, model 3 in table S3). Nestlings were significantly more likely to lower their heads when hearing the warbler sound after training compared with before training (GLMM: estimate = 2.03, s.e. = 0.77, *z* = 2.63, *p* = 0.008; figure 2c and electronic supplementary materials, model 3 in table S3 and video S1). In contrast, nestlings did not differ in the tendency to lower their heads when hearing the rosefinch sound (control treatment without alarm calls) after training compared with before training (GLMM: estimate = −0.78, s.e. = 0.74, *z* = −1.06, *p* = 0.29; figure 2c and electronic supplementary material, video S1). After training, nestlings were more likely to lower their heads when hearing the warbler sound compared with the rosefinch sound (GLMM: estimate = 1.52, s.e. = 0.65, *z* = 2.33, *p* = 0.02; figure 2c and electronic supplementary material, model 7 in table S4). However, there was no interaction between the playback treatment and the day of the post-training test (GLMM: estimate = 0.28, s.e. = 0.93, *z* = 0.30, *p* = 0.77; figure 2c and electronic supplementary material, model 7 in table S4), indicating that the propensity to lower the head remained stable over the 2 days following training.

Contrary to expectations, for nestlings in the rosefinch group (treatment with alarm calls), the interaction effect between treatment and stage was not significant (GLMM: estimate = −1.22, s.e. = 1.11, *z* = −1.10, *p* = 0.27; electronic supplementary material, model 4 in table S3). However, nestlings in the rosefinch group also tended to show a greater variation in the probability of lowering their heads in response to the rosefinch sound compared with the warbler sound after training than before training (figure 2d). They were more likely to lower their heads when hearing the rosefinch sound after training compared with before training (GLMM: estimate = 1.57, s.e. = 0.69, *z* = 2.27, *p* = 0.023; figure 2d and electronic supplementary material, model 4 in table S3 and video S2). In contrast, nestlings did not differ in the likelihood of lowering their heads when hearing the warbler sound (control treatment) after training compared with before training (GLMM: estimate = 0.35, s.e. = 0.88, *z* = 0.40, *p* = 0.69; figure 2d and electronic supplementary material, video S2). After training, nestlings were also significantly more likely to lower their heads in response to the rosefinch sound compared with the warbler sound (GLMM: estimate = 1.90, s.e. = 0.74, *z* = 2.57, *p* = 0.01; figure 2d and electronic supplementary material, model 8 in table S4). Furthermore, there was no interaction between the treatment and the day of the post-training test (GLMM: estimate = −0.08, s.e. = 0.90, *z* = −0.09, *p* = 0.93; figure 2d and electronic supplementary material, model 8 in table S4), again suggesting that the probability of lowering the head remained consistent over the 2 days following training.

## 4. Discussion

Our results demonstrate that Daurian redstart nestlings can socially learn to recognize unfamiliar sounds as alarm signals by associating them with conspecific alarm calls. Before training, only a few nestlings responded to the unfamiliar sounds, and there was no difference in their responses between the two sounds. After training, however, nestlings were much more likely to respond to the training sound than to the control sound. Moreover, significantly more nestlings responded to the training sounds, but not to the control sounds, after training compared with before training. These findings suggest that redstart nestlings learned to identify specific unfamiliar sounds as alarm signals, rather than displaying a generalized heightened alertness. Moreover, the nestlings retained their learned responses on the second test day, suggesting stability of the learned recognition.

It has been shown that adult birds can learn to eavesdrop on novel alarm calls by associating them directly with the presence of predators (asocial learning) [17], or indirectly with known alarm calls, without having to see the callers or the predator (social learning) [16]. However, whether nestlings can learn to eavesdrop on heterospecific alarm calls remains unclear. To our knowledge, only two studies have tested the idea, and with mixed results [19,35]. A study on great tits, *Parus major*, exposed nestlings to a non-threatening novel sound together either with conspecific mobbing calls (training sound) or with another non-threatening novel sound (control sound), and found no difference in nestlings’ responses between the treatments, suggesting that great tit nestlings could not learn to respond to novel alarm signals through associative learning [35]. However, after fledging, the nestlings showed increased scanning behaviour in response to the training sound, but not to the control sound, indicating that they had formed a link between the heterospecific and conspecific alarm calls through associative learning. In contrast, a study on white-browed scrubwrens, *Sericornis frontalis*, found that young nestlings did not initially respond to alarm calls from New Holland honeyeaters, *Phylidonyris novaehollandiae*, and superb fairy-wrens, *Malurus cyaneus*, but did respond appropriately to these calls as they aged [19]. The authors suggested that the nestlings could have learnt to recognize the calls [19]. However, an alternative explanation is the delayed development of innate responses to these calls [47]. Both the honeyeaters and fairy-wrens breed sympatrically with the scrubwrens and share similar nest predators [19], so scrubwren nestlings may have innate responses to their alarm calls, as they do to the alarms of brown thornbills, *Acanthiza pusilla* [19]. Even with innate recognition, young nestlings may not be physiologically mature enough to develop the sensory system required to recognize honeyeater or fairy-wren calls ([47,48], but see [19]). In the present study, we used sounds from brown-flanked bush warblers and common rosefinches, both of which breed allopatrically with the redstarts, implying that it is highly unlikely that redstart nestlings would have an innate response to these sounds. Although a few nestlings responded to the sounds in the pre-training tests (figure 2), these responses are likely due to wariness (see below). In stark contrast, after training, nestlings often responded to the training sound, but not to the control sound, suggesting that the nestlings learned to recognize the novel sounds through associative learning, i.e. by associating the novel sounds with conspecific alarm calls.

Innate recognition enables young animals to respond quickly to threats, minimizing the need to learn about predators through trial and error [47]. However, given the substantial variation in alarm calls among species, innate responses may be limited, especially in species with widely distributed breeding areas such as Daurian redstarts. In these cases, recognition through learning can help individuals develop appropriate responses to a broader range of locally relevant, novel alarm signals and thereby to fine-tune their antipredator behaviours to a changing environment [7,25]. Moreover, because it does not require direct exposure to predators, associative learning reduces the costs associated with trial-and-error learning in nestlings [25,47].

It is well established that the ability to eavesdrop on heterospecific alarm calls can develop early in life [19,49,50]. For instance, fledgling white-browed scrubwrens acquire appropriate responses to New Holland honeyeater alarm calls on territories where the honeyeaters are common, but fail to respond to those alarm calls in areas without honeyeaters [49]. Similarly, infant vervet monkeys, *Cercopithecus pygerythrus*, learn to respond to the mobbing calls of superb starlings, *Lamprotornis superbus*, more rapidly in areas where starlings are abundant [50]. While these examples highlight the role of early experience, the mechanisms underlying this learning—whether direct experience or social learning—remain unclear. Here, we demonstrate that Daurian redstart nestlings can socially learn to eavesdrop on heterospecific alarm calls by forming an acoustic association with conspecific alarm calls.

In conclusion, this study experimentally demonstrates that Daurian redstart nestlings can socially learn to recognize unfamiliar sounds through a process of acoustic–acoustic association. Notably, the learning process is rapid, with nestlings showing a marked change in behaviour after exposure to six unfamiliar heterospecific sounds in association with conspecific alarm calls. Overall, our findings suggest that social learning can take place at a young age. Young animals are not simply passive victims of predation; instead, they can use social (or associative) learning to actively engage in behaviours to reduce predation risk.

## Data Availability

Data and R code used in this study are available from the Dryad Digital Repository [51]. Supplementary material is available online [52].
